# Impact of using different-sized touch keyboards on free-text keystroke dynamics authentication in the Arabic language

**DOI:** 10.1038/s41598-022-20099-6

**Published:** 2022-09-23

**Authors:** Suliman A. Alsuhibany, Afnan S. Almuqbil

**Affiliations:** Department of Computer Science, College of Computer, Qassim University, Buraydah, 51452 Saudi Arabia

**Keywords:** Engineering, Electrical and electronic engineering

## Abstract

Authentication using keystroke dynamics (KD) has become an active research area due to its usability and security aspects. Nowadays, the scale of touch keyboard use has expanded to include most modern devices. Although KD typically focuses on a single device at a particular time, authentication systems are adjustable within their environments, as these systems’ users frequently switch between multiple devices. Thus, this paper assesses users’ typing behavior on different tablet devices with varying touch keyboard sizes. In particular, we empirically assess whether the validation results from free-text KD authentication vary depending on the touch keyboard size. The results reveal interesting research directions for future feasibility studies on changing the dynamic keystroke typing-pad effect on user-security and trust-authentication analysis.

## Introduction

Data protection has become crucial in recent years, associated with the increasing use of online transactions. Consequently, numerous researchers have sought to provide strategies to improve information security in various fields, such as banking^1^ and hospitality^2^. Many researchers have also suggested new techniques to achieve more secure online authentication^3–5^. Furthermore, many of the proposed techniques evaluate smart device applications’ personal privacy^6^.

User authentication also is a very challenging domain to implement in a safe and reliable way. Commonly used authentication approaches gradually have evolved with the emergence and increased popularity of new and innovative devices within the consumer market. However, both traditional and recent security measures suffer from some significant usability and security hindrances. Various authentication techniques have been developed to ensure security in applications, such as facial recognition, personal identification numbers (PINs), and fingerprint scanning^7^.

Although these mechanisms’ functionality offers a certain level of security, these security standards also easily can be compromised^8^. For instance, passwords may be leaked, shoulder-surfed, or broken using password-defying software tools. Furthermore, passwords often are shared or written down for future use, representing an additional security threat^9,10^. Additionally, the fingerprint method is vulnerable to being spoofed through imitation of the fingertip structure, as generated through a concealed fingerprint^11^. Even smartphones often fail to recognize fingertips and, thus, require multiple access attempts. Similarly, facial recognition can be spoofed, as people can use pictures, videos, or 3D masks to forge a user’s face to access their data^12^.

These authentication mechanisms, along with being highly susceptible to illegitimate use, also require hardware to support their services, ultimately adding to the device’s cost. Using an alternative authentication mechanism is ideal in such situations. Specifically, these alternatives not only should have a low cost, but also offer some usability and security levels, e.g., keystroke dynamics authentication (KDA)^13^.

KDA heavily relies on behavior-based characteristics as its authentication mechanism, particularly the rhythm and manner in which users type and use the keyboard. Many advantages are associated with behavioral authentication systems that counter deficiencies in traditional authentication mechanisms. First, generating similar movement patterns can be difficult to imitate practically. Even if the pattern is imitated, the human body’s relative structure––e.g., height, shape, and finger orientation on the touchscreen––will be different and result in differences in movement patterns. Furthermore, the built-in physical sensors within digital devices represent features that can be detected easily––even small differences between these characteristics––and subsequently block access. Moreover, every person has a unique way in which they input data. Although an unauthorized person may copy a password, an authentic user’s touch style, pattern, and type cannot be imitated easily^14^. Furthermore, two main keystroke text types exist: fixed and free text. The former refers to predefined text that should be entered during the log-in stage, while the second one refers to entering any text during the log-in stage. This study solely considers free text because it has many advantages over fixed text. For example, with free text, the pattern does not depend on the typed text; therefore, the user need not memorize a password. Furthermore, the authentication in free text occurs not only during log-in, but also afterward, in which users inside the system continuously are monitored based on their typing behavior. Furthermore^15^, proved that free text provides more accuracy than fixed text. Consequently, we chose free text as our study’s baseline.

Even though many KD authentication studies on physical keyboards have been conducted, little attention has been paid to touch KD authentication on virtual keyboards^16^. Moreover, with the increasing trend in people being accustomed to using multiple devices with different virtual keyboard sizes, taking a step in this direction by using techniques, e.g., touch dynamics, to improve security across multiple devices is necessary.

The Arabic and English languages are not the same, as Arabic is a Semitic language belonging to the Afro-alphabet language family, whereas English is a Germanic language from the Indo-European language family^17^. Moreover, previous studies^16,18^ have analyzed Arabic language free-text KD. These studies’ results were interesting in terms of both false acceptance rate (FAR) and false rejection rate (FRR).

Therefore, this paper examines how users’ typing behavior on one device is significantly unique relative to the same user’s typing behavior on other devices with different touch keyboard sizes. To examine this, an experimental study was conducted using three different touch screen sizes: 8-inch; 10.8-inch; and 12.6-inch. Moreover, the random forest (RF) classifier was selected and implemented with the aim of determining whether using different touch keyboard sizes impacts Arabic language touch keystroke authentication systems. This experiment’s results demonstrated that touch keystroke authentication systems can achieve the same accuracy level by switching between devices with different touch keyboard sizes.

Thus, this study contributes to the Arabic keystroke authentication system (AKA) field as follows.

- Investigation of the impact of screen size on touch keystrokes and AKA accuracy, which is, to the best of our knowledge, the first such study to do so.
- Utilization of Arabic script concerning screen size’s impact on touch keystroke and AKA accuracy, considering that the Arabic and English languages are completely different^17^.

The rest of this paper is structured as follows: Related works are discussed in Section “Related works”. The methodology is described in Section “Methodology”. Section “Experimental study” explains the experimental study. The results are presented and discussed in Section “Results” and “Discussion”. Section “Conclusion and future works” concludes the paper and offers suggestions for future research directions.

## Related works

Several recent studies have examined touch keystroke authentication systems, but to the best of our knowledge, none of them examined whether using different touch keyboard sizes impacts Arabic language touch keystroke authentication systems. We aim to fill this literature gap. Thus, in this section, we discuss relevant extant studies.

First, Draffin et al.^19^ examined a passive authentication approach in which a non-authorized user can be identified passively by modelling the micro-behavior of the user’s interactions with their devices. The results from evaluating this approach revealed that the FAR was 32.3%, while the FRR was 4.6%.

The mobile’s sensors were utilized in Gascon et al.^20^, in which the user’s biometric behavior is exploited while interacting with the mobile devices. This can authenticate users continuously with high precision. Moreover, the FAR and FRR were 92% and 1%, respectively.

A study by^21^ experimentally examined a free-text keystroke authentication system using different devices. The results indicated that the touch keyboard had an acceptable authentication accuracy level, with an 8.9% equal error rate (EER). Likewise^22^, proposed a mobile device authentication approach based on accelerator, time, free text, and coordinator. This approach was evaluated through three different mobile sensors, and the results elicited an EER of less than 1%. Furthermore, Alsuhibany and Almuqbil^15^ examined the Arabic KD authentication (AKDA) technique’s influence on touch keyboards. The examination indicated that AKDA positively impacts security level through higher accuracy, with a 0% EER. Table 1 summarizes these studies on free KDA for touch keyboards.

**Table 1:** Summarized studies on free KDA for touch keyboards.

Study	Features	FAR, FRR, or EER
^19^	Time, pressure, gyroscope, coordination, and size	FAR = 14%, FRR = 2.2%
^20^	Time, accelerometer, gyroscope, and oriented sensor	FAR = 92%, FRR = 1%
^21^	Time	EER = 8.9%
^22^	Time, acceleration, and coordination	EER = 0%
^15^	Time, acceleration, gyroscope, pressure, and coordination	EER = 0%

Furthermore, another study conducted by Zaidan et al.^23^ argued that typing time via KDA varies depending on the hardware used, i.e., they found that average personal computer keyboard typing time is faster than that of other keyboards (e.g., handheld devices’ keyboards), mainly because the user employs both hands when typing without the need to change the layout for typing, e.g., special characters. Alternatively, keyboards on handheld devices did not register the worst time for particular layouts of mobile phone keyboards.

Furthermore^24^, focused on assessing user identification’s influence under optimal (i.e., similar types of keyboards) and non-optimal (i.e., different types of keyboards for enrollment and testing) conditions. The different types were desktop and laptop keyboards. When similar keyboards were used for enrollment and testing, the identification accuracy rate was 99.5% for laptop keyboards and 98.3% for desktop keyboards. However, this rate decreases considerably when the users employed different types of keyboards for enrollment and testing. This finding corroborates previous results presented by Matsubara et al.^25^, who observed that using different keyboards to type Japanese text impacts system accuracy, except for individuals who are skilled typists, whose accuracy rate was 99%.

Alsuhibany et al.^17^ reported similar findings, in which two different keyboards were used (i.e., MacBook Pro laptop and HP laptop keyboards). They found that the two keyboards’ layouts greatly influenced system performance during the log-in and sign-up phases, but these findings were based on physical keyboards.

However, recent research has found that keyboard type makes no difference on typing results. One such study, by Belman and Phoha^26^, examined users’ typing behavior across different devices with various keyboards, e.g., tablet, phone, and desktop. They extracted the relationship between the various factors and pointed out that when users switch between devices, they can be authenticated to over a multi-device environment.

Based on the aforementioned previous research, our study aimed to determine whether the validation results vary based on touch keyboard size. Thus, this paper uses the same keyboard layout, but in different touch keyboard sizes.

## Methodology

This section lays out the chosen methodology for the experiment, particularly data collection, extraction of features, preprocessing, and classification. *Notably*, *all experimental protocols and methods were approved by the Department of Computer Science*, *College of Computer*, *Qassim University*.

### Data collection

To gather data for the research, a touch keyboard authentication system implemented in Alsuhibany and Almuqbil^15^ was utilized. The system processes recordings of raw data, which then are extracted when a user touches keys on the keyboard. Furthermore, when the given text is written and submitted, the system automatically generates a user profile, which is stored within the device’s local database. Moreover, this profile comprises four features: time stamps; acceleration; coordination; and pressure. These features are detailed in the following subsection.

### Extraction of features

The reason behind selecting all the aforementioned features is mainly due to their efficient performance in the state-of-the-art approaches. For instance, several studies^27–29^ have demonstrated excellent performance with these features in validating KDA systems. Each feature’s extraction is explained below.

#### Time stamps

**Figure 1:**
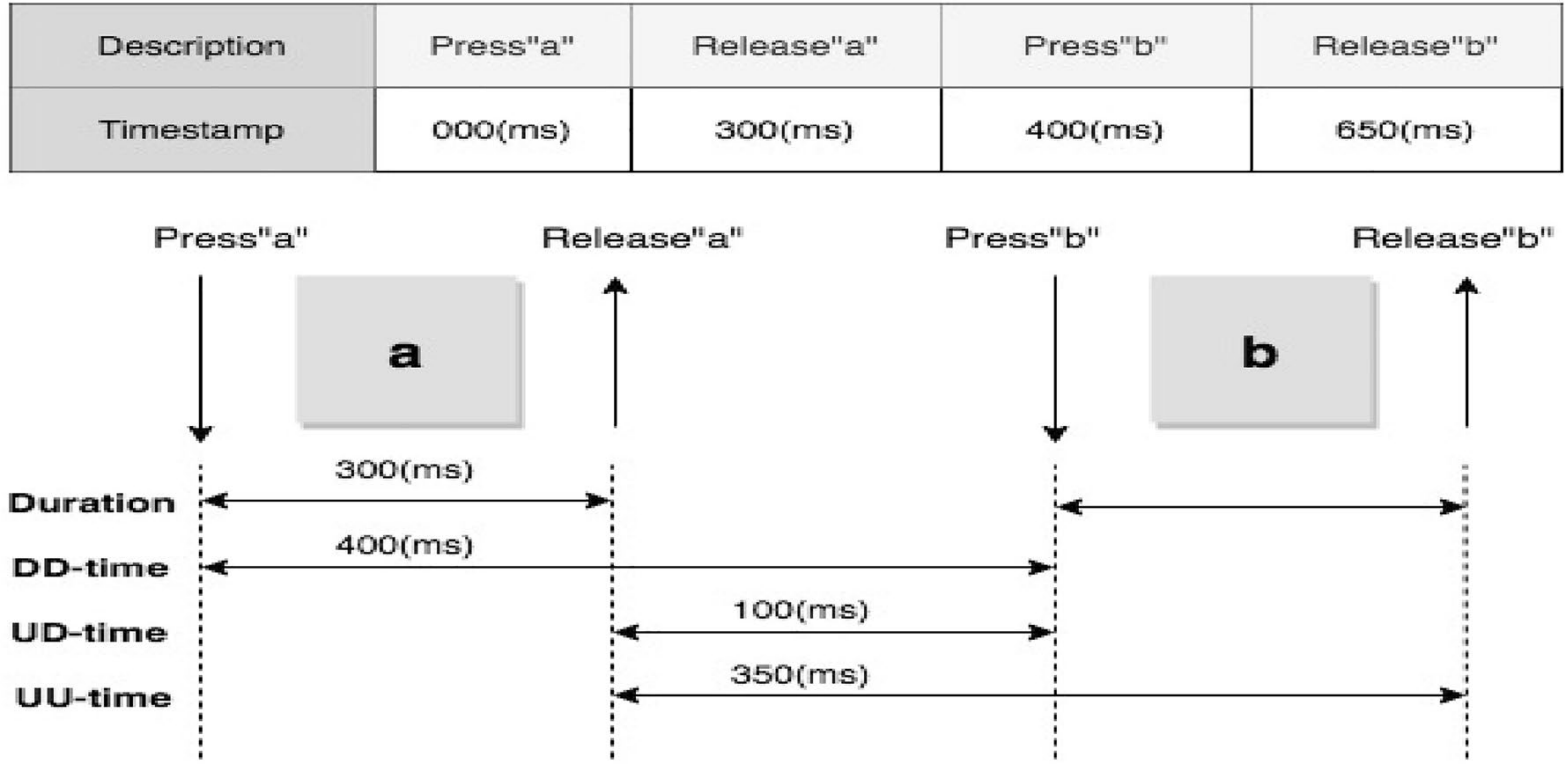
KDA timing features (Alsuhibany and Almuqbil^15^).

These are attained from two actions on the keyboard: depression and release. The former refers to the time stamp recorded at the time when the key is touched and held down (D), while the latter refers to the time stamp recorded at the time when the key is released (U). Precise timing features can be attained through each event’s time stamps, as depicted in Fig. 1.

Furthermore, these timing features in Figure 1 are described as follows:

- *Hold time (Down-Up)* The key is touched until released.
- *Latencies/Flight time* Down-Down (DD) time or Up-Up (UU) time represents the time between two sequential keys touched.
- *Di-graph duration* Up-Down (UD) time refers to the elapsed time between release of the first key and depression of the second key.

#### Acceleration

This feature calculates the device’s acceleration (m/s^2^) on three axes: lateral x-axis; longitudinal y-axis; and vertical z-axis.

#### Coordination

The coordinate values of the horizontal x-axis and vertical y-axis are extracted when the key is touched on the touchscreen device.

#### Pressure

The pressure force is returned when the user touches the key on the touchscreen. The returned pressure measurements are of an abstract unit, ranging from 0 (no pressure) to 1 (normal pressure).

#### Combination of features

All the described features are concatenated to get combined features.

### Preprocessing

We used the same approach applied in Alsuhibany and Almuqbil^15^ to trace outlier data pertaining to every user by utilizing an interquartile range, i.e., to improve classification performance, the outlier data were excluded before the classifiers were fed.

### Classification

We undertook experimental studies for various scenarios in which each scenario had corresponding data sets. In particular, RF classifiers were used to perform verifications using a Python programming language library––the Scikit-Learn library. Notably, the RF was adopted because it has demonstrated good results in a variety of related research^15,30^.

## Experimental study

We conducted an experimental study to test our aim (i.e., determining whether using different touch keyboard sizes impacts Arabic language touch keystroke authentication systems) via the developed application. The following sections explain each part of the experiment in detail. *Notably, all experiments were performed in accordance with Computer Science Department Scientific Committee standards. Also, all experiments were approved by the Ethics Committee in the Computer Science Department, College of Computer, Qassim University*.

### Experiment setup

This subsection discusses details on the experiment’s design, participants, systems, and materials used.

#### Experimental design

A within-subjects controlled laboratory experimental study method was chosen to ensure that the data collection process was free of any interference or disruptions. Considering that we tested three different touch screen sizes––8-inch, 10.8-inch, and 12.6-inch––the experiment was divided into three sessions: During each session, the user was asked to type Arabic text into one of three Android devices. Details of these devices are provided in Table 2. Each session took approximately 15 min. Considering that the same users participated in each session, 10-min breaks were provided between sessions. It has been determined in the pilot study that these breaks are convenient. The participants completed these sessions’ activities in the following order: Tablet A; Tablet B; and Tablet C. Table 3 summarizes the collected data.

**Table 2:** Selected devices’ information.

	A	B	C
Device’s name	Huawei MatePad Pro	Huawei Media Pad M6	Huawei MatePad T
Display size	12.6-inch	10.8-inch	8-inch
Dimensions	184.7 × 286.5 mm	170 × 257 mm	121.10 × 199.70 mm

**Table 3:** Summarizing collected data.

Number of sessions	Three
Each session’s length	15 min
Devices	Tablet A, Tablet B, and Tablet C
Keystroke text type	Free-text keystroke
Text in log-in session	حسن الظن يوحد قوة الروح وقوة الجسد ومن استقرار الروح تزدهر الصحة النفسية التي ترتبط غاية الارتباط بقدرة الشخصية على التوافق مع نفسها ومجتمعها الذي نعيش فيه وهذا يقود للتمتع بحياة ساكنه سوية مفعمة بالحماس
Text in sign-up session	الاخلاق في الدين الإسلامي ماهي الا مجموعة آداب وقيم المنظمة للسلوك الإنساني لتنظيم حياة الانسان وتحديد علاقته بغيره بحيث يحقق الغاية من وجوده في هذا العالم من الواجب التحلي بها وقد حث الإسلام عليها لكونها من الصفات التي تميز انسان عن غيره ولكونها سبيل يقرب الفرد لربه
Number of participants	40

#### Participants

First, informed consent was obtained from all participants, which ended up being 40 users, all of whom took part in the experiment over a two-week period. The participants were between 17 and 22 years old, possessed varying typing skills, and had Arabic as their native language. Furthermore, most users were quite familiar with the medium-size device (i.e., Tablet B), while some had experience with other touch screen sizes.

#### Materials

The participants were required to type two texts: one during the log-in phase, which had 203 characters with spaces, and the other during the sign-up phase, which had 267 characters with spaces.

#### System

**Figure 2:**
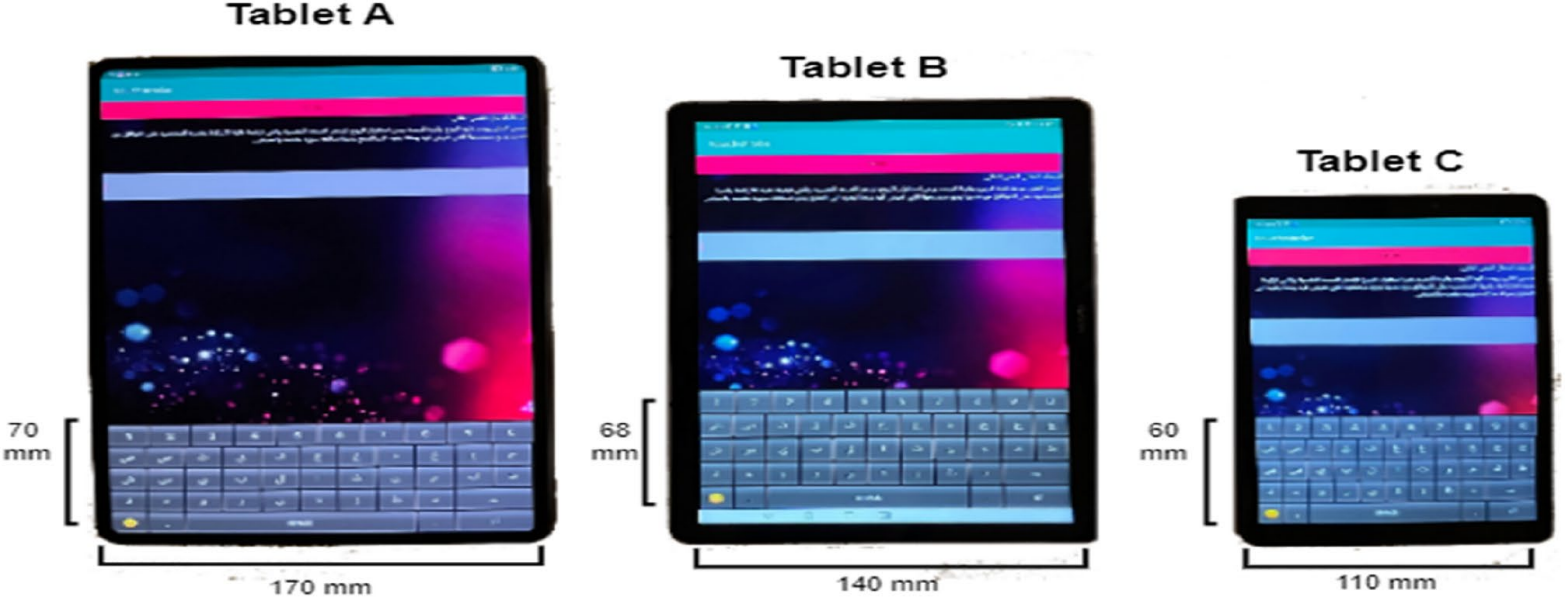
Each tablet’s keyboard size.

The system developed in prior research by Alsuhibany and Almuqbil^15^ was employed in this study to collect data, as it was installed on three different Android touch screen devices with different sizes. Figure 2 depicts the keyboard layout for each device used. The data were stored in an SQLite database in each device. When the users completed the sign-up step successfully, they automatically were directed toward the log-in interface.

#### Evaluation matrix

**Figure 3:**
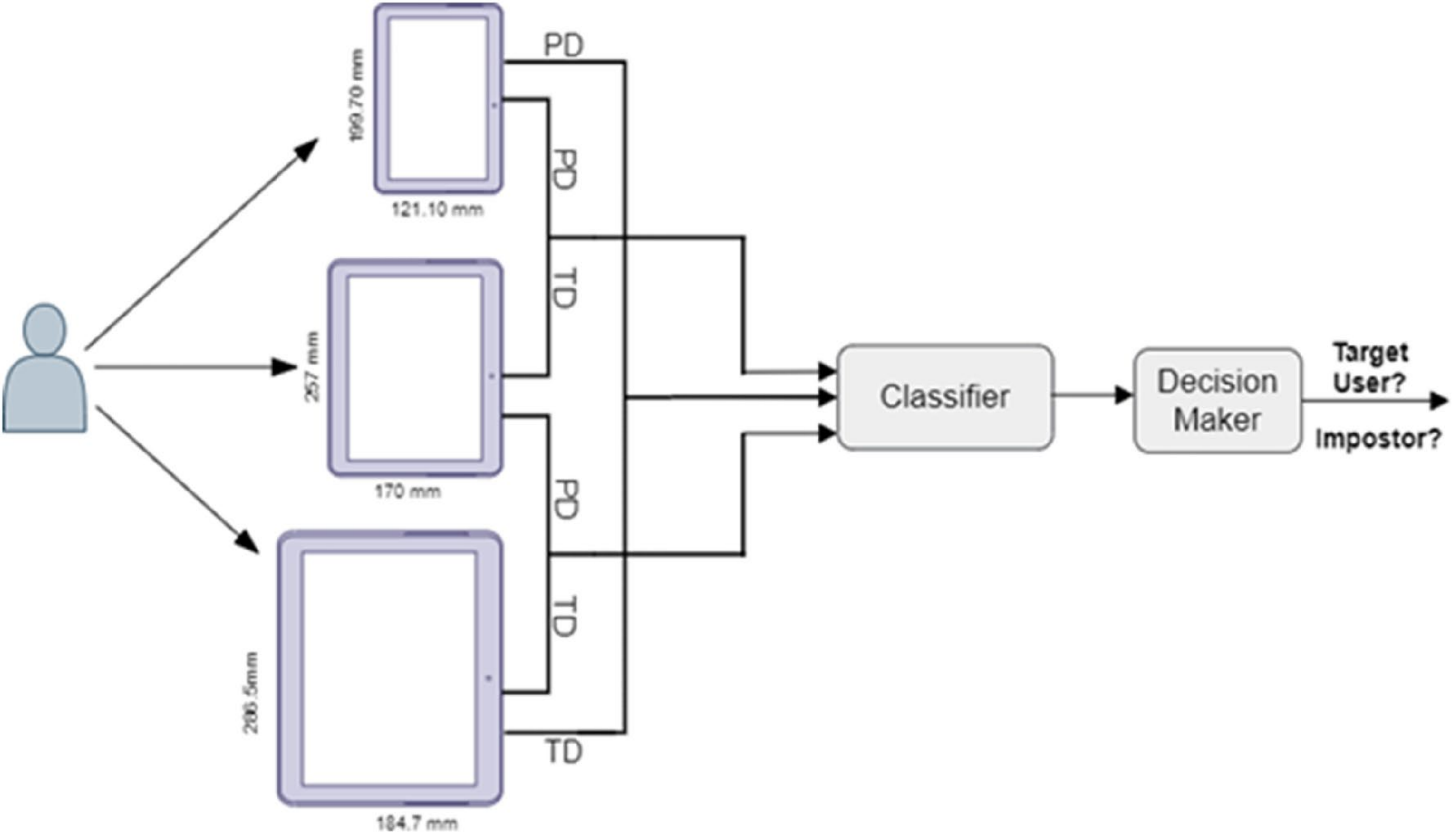
KDA system using different devices (*PD* refers to profile data, while *TD* refers to test data).

Three main metrics were used in our experiment: FRR; FAR; and EER. Specifically, FRR refers to the ratio of authorized users who are rejected and denied access to the system incorrectly. Conversely, FAR refers to the ratio of unauthorized users who can access the system. EER previously has been explained in section “Related works”. Thus, we calculated the FRR and FAR metrics as follows. For FAR, the experiment compared the user’s test data, which were stored on the first tablet device, with the remaining users’ profile data stored in the second tablet. FRR was calculated by comparing the user’s test data, which were stored in the first tablet, with those of the user profiles stored in the second tablet, as shown in Fig. 3. The EER formula is stated in Eq. (1):

1 $$EER=\frac{FAR+FRR}{2}$$

### Experimental procedure

In this section, we explain how we conducted the experiment, including the instructions given to the participants, the procedure for conducting the experiment, and the data collection process.

#### Instructions to participants

At the beginning, the users were instructed that the experiment focused on users’ ability to write the given text in regular form. The users were instructed that the mobile phones should be switched off or placed in silent mode so that they would not be interrupted during the process of writing the text. The users were instructed to sit in a chair while holding the tablet in their hands to help achieve greater accuracy, as stated in Roh et al.^29^.

Before starting the experiment, the users were given some time during which they could try out the system to familiarize themselves with it. During the writing portion, users were instructed to use the spacebar or backspace keys wherever required. At the end of the writing portion, a thank-you message appeared to confirm that the experiment was completed.

#### Experimental study procedure

To authenticate the participants used KDA on the tablet devices, the typing process was implemented in two phases. The first was the enrollment (sign-up) phase, also commonly known as a profile-building phase, i.e., the participant’s typing rhythm on the touch keyboard was recorded over several trials to recognize the most close typing behavior profiles. Furthermore, the user during the second phase was instructed to type the log-in text, which then was matched with another device’s profile text.

Once these phases were completed, we introduced three scenarios to facilitate our investigation into users’ typing behavior on different tablet devices with varying touch keyboard sizes. These scenarios were implemented as follows:

- *Scenario 1* We compared the user’s profile data with the user test data in Tablet A, then compared the user profile in Tablet A with the user’s test data in Tablets B and C.
- *Scenario 2* We compared the user’s profile data with the user test data in Tablet B, then compared the user profile in Tablet B with the user’s test data in Tablets A and C.
- *Scenario 3* We compared the user’s profile data with the user test data in Tablet C, then compared the user profile in Tablet C with the user’s test data in Tablets A and B.

#### Collected data

A user profile was created in each device once a user attempt was completed successfully. This profile included time, accelerometer, coordination, and pressure data. Several metrics for each feature (i.e., mean, maximum, and minimum) were stored in another table. Therefore, the final data set comprised 21 features: Avg.HoldTime; Max.HoldTime; Min.HoldTime; Avg.UD; Max.UD; Min.UD; Avg.DD; Max.DD; Min.DD; Avg.UD; Max.UD; Min.UD; Avg.accelerometer; Max.accelerometer; Min.accelerometer; Avg.Pressure; Max.Pressure; Min.Pressure; Avg.coor; Max.coor; and Min.coor.

## Results

**Figure 4:**
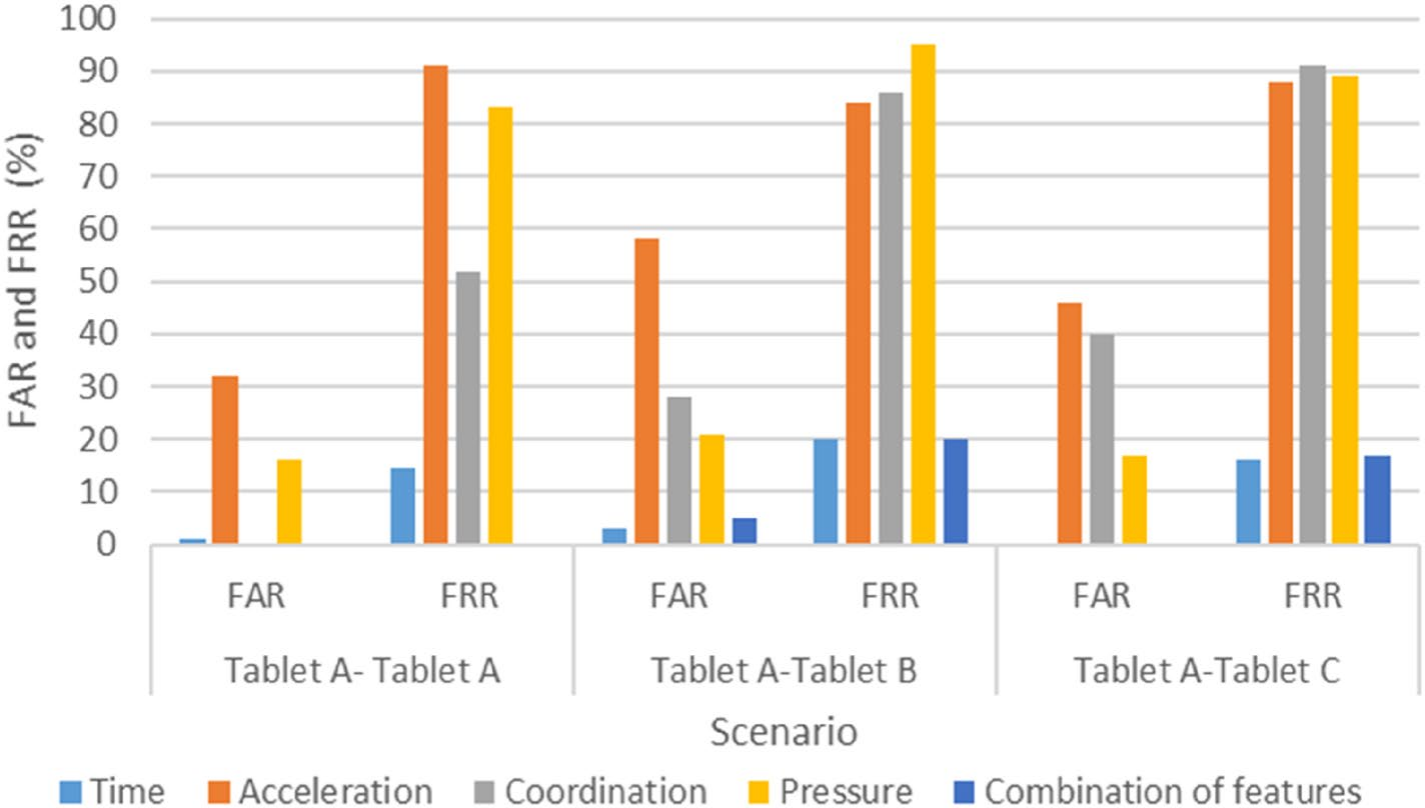
FAR and FRR of Scenario 1 (Tablet A–Tablet A, Tablet A–Tablet B, and Tablet A–Tablet C).

**Figure 5:**
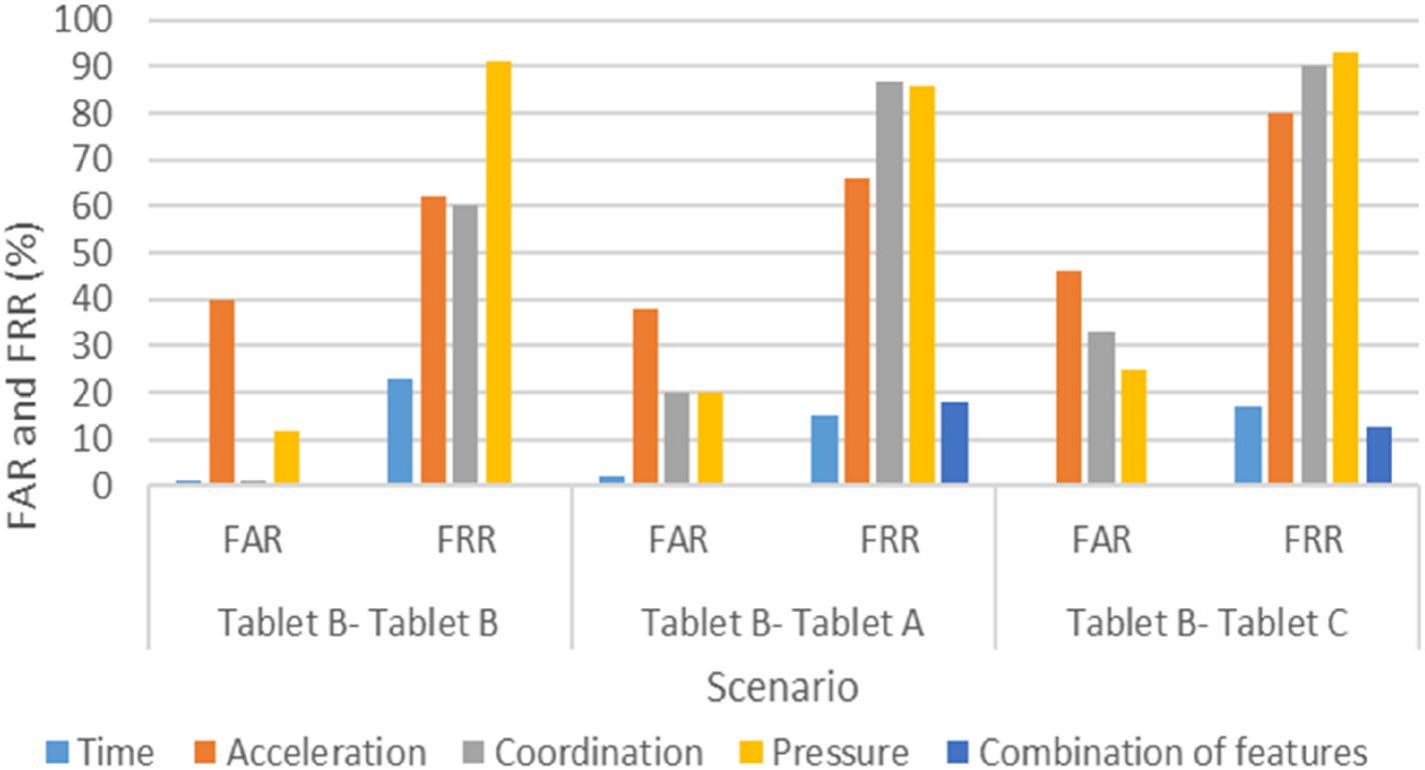
FAR and FRR of Scenario 2 (Tablet B–Tablet B, Tablet B–Tablet A, and Tablet B–Tablet C).

**Figure 6:**
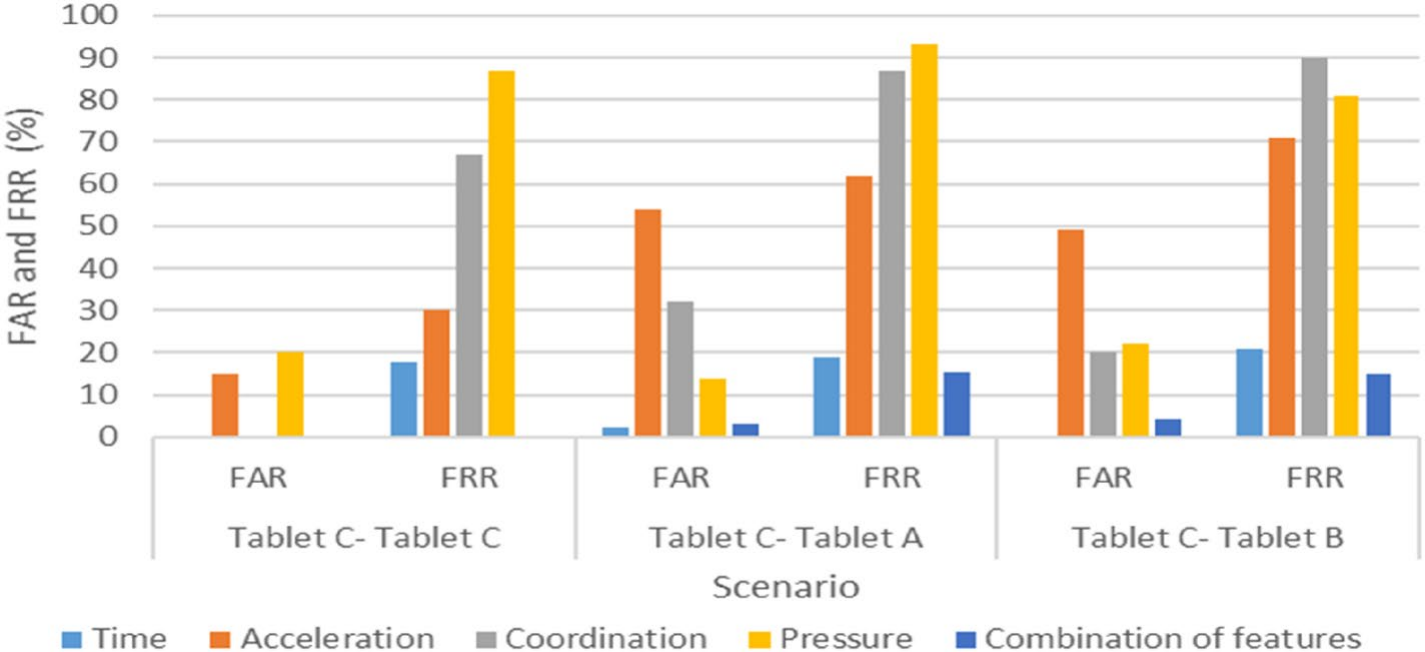
FAR and FRR of Scenario 3 (Tablet C–Tablet C, Tablet C–Tablet A, and Tablet C–Tablet B).

All participants successfully completed the experiment in two weeks. Figures 4, 5, and 6 present each scenario’s FAR and FRR, and Table 4 lists the three scenarios’ EERs.

**Table 4:** Each feature’s error rates.

Training data	Testing data	Features				
		EER %				
		Time (%)	Acceleration (%)	Coordination (%)	Pressure (%)	Combination of features (%)
**Scenario 1**						
Tablet A	Tablet A	6	61	26	48	0
Tablet A	Tablet B	11	70	55	62	12
Tablet A	Tablet C	9	68	60	53	9
**Scenario 2**						
Tablet B	Tablet B	10	50	30	50	0
Tablet B	Tablet A	8	53	55	54	9
Tablet B	Tablet C	10	64	62	58	6
**Scenario 3**						
Tablet C	Tablet C	9	21	33	55	0
Tablet C	Tablet A	12	56	59	47	8
Tablet C	Tablet B	10	59	56	51	7

In particular, the results indicate that the time feature recorded lower FAR and FRR values when the user switched between devices, and when using the same device. The average FAR and FRR values were 1.1% and 18.2%, respectively, whereas the most effective FRR and FAR values were 15% and 0%, respectively. Furthermore, the combination of all features also produced high accuracy, but in the case of switching between devices, the improvement was insignificant relative to the large data volume resulting from acceleration, coordination, and pressure features. Therefore, Figs. 4, 5, and 6 illustrate that by using the time feature, the KDA approach is applicable when using different keyboard sizes. Thus, users need not be restricted by a particular device when they authenticate remotely.

Interestingly, when switching between multiple devices, coordination and acceleration had the worst performance, with acceleration and coordination reaching an EER of 70% and 62%, respectively, when moving from Tablet A to Tablet B and from Tablet B to Tablet C.

Likewise, with respect to using test and profile data from the same device, we observed that the acceleration feature became even worse when the keyboard size expanded, wherein the FRR score in the acceleration feature tripled compared with that of the smallest tablet (i.e., from 30 to 91). The EER score in the coordination feature indicated some improvement after an increase in key size, ranging from 33% EER when using the small device to 26% EER when using the large device.

## Discussion

The experimental results generally revealed that using three different touch keyboard sizes does not impact performance in the system when using the time feature, likely because all keyboards have the same layout. These findings correspond with those by Belman and Phoha^26^, who obtained satisfactory results with mean accuracies of 99.31%, 99.33%, and 99.12% for desktop-phone, desktop-tablet, and tablet-phone relationships, respectively. As shown in Table 5, Belman and Phoha^26^ used three devices with different keyboard sizes, RF classifiers, and time features. However, Alsuhibany et al.^16^ demonstrated that a considerable change occurs in the system’s accuracy when different keyboards are used, even with the same time features and Arabic text inputs. This investigation’s results indicated that EER was 34% using the first keyboard and 32% using the second keyboard.

**Table 5:** A comparison of our system results and the results from^16^ and ^26^.

Study	Methodology	Features	Classifier	Result
^16^	Used two different keyboard layouts The first laptop was a MacBook Pro The second laptop was an HP	Time features	Euclidean distance	34% EER using MacBook Pro for testing data and HP for training data 32% EER using HP for testing data and MacBook Pro for training data
^26^	Used three devices with different keyboard sizes (desktop, tablet, and phone)	Time feature	RF classifier	Accuracies of 99.31%, 99.33%, and 99.12% for relationships between desktop phone, desktop tablet, and tablet phone, respectively
Our study	Used different tablet devices of varying sizes (i.e., 8-inch, 10.8-inch, and 12.6-inch)	Time features acceleration coordination pressure and a combination of features	RF classifier	The best results were 0% EER when the participants used the same device and 6% EER when they used different devices

Notably, when the same keyboard sizes were used between the enrollment and testing phases, acceleration and coordination generated interesting results. For acceleration, the accuracy rate was quite high when a small tablet device was used, as shown in Fig. 6, possibly because when the device’s size increases, handling the device becomes more challenging. However, with virtual keyboards that have a key size of less than 16 mm, it is generally too small for touch screen typing, resulting in slower typing speed, as Kim et al.^31^ reported. Furthermore, our findings corroborate those of previous research in^22^, namely that a larger key size leads to an increase in the results’ accuracy, i.e., as device size increases, the coordinates’ data accuracy also increases. Accordingly, a possible trade-off may occur between acceleration and coordination, with device size positively impacting acceleration, but negatively impacting coordination. Thus, this might need to be investigated to find the recommended size, which will be one of our future works.

Generally, it was found that the combination of varying features produced highly accurate results, followed by the time features when using the same device and changing between devices. However, coordination was more effective in the case of using the same device during the log-in and sign-up sessions. The acceleration produced the best result when the smallest device was used (i.e., Tablet C) during both sessions. However, the worst feature was pressure, which did not produce acceptable results in all cases.

Furthermore, during the experimental process, although most of the participants were familiar with medium-size tablets (i.e., Tablet B), the results from using this tablet did not indicate higher accuracy than that of the other two devices. This indicates that familiarity with a particular size keyboard does not generate a bias in the results when a different-sized keyboard is used, but the keyboard layout remains the same.

## Conclusion and future works

This paper investigated the impact of using three different sizes of touch keyboard layouts in the user authentication process when typing Arabic free text. In particular, an experimental study was conducted by applying three different sizes of touch-based digital devices. The experiment’s results demonstrated that using the time feature for KD-based authentication offers a feasible technique for multi-device environments, i.e., we achieved average FAR and FRR scores of 1.1% and 18.2%, respectively.

A significant change in the system’s accuracy was observed when the acceleration and coordination features with different keyboard sizes were used. These findings may be useful when authenticating users in online applications that allow for switching between multiple devices.

Future studies also can be built on this one by using different keyboard layouts. In the future, we will use additional features to evaluate whether different features enhance the results for devices with different sizes.

## Data Availability

The datasets used and analysed during the current study available from the corresponding author on reasonable request.
